# Screening of Cytotoxic and Genotoxic Activities of Subcritical Water Extracts from *R. damascena* and *R. alba* Flowers

**DOI:** 10.3390/molecules30214294

**Published:** 2025-11-05

**Authors:** Tsvetelina Gerasimova, Svetla Gateva, Gabriele Jovtchev, Ana Dobreva, Milka Mileva, Zlatina Kokanova-Nedialkova, Milena Gospodinova, Tsveta Angelova, Paraskev Nedialkov

**Affiliations:** 1 Institute of Biodiversity and Ecosystem Research, Bulgarian Academy of Sciences, 2 Gagarin Str., 1113 Sofia, Bulgaria; cvetij@yahoo.com (T.G.); spetkova2002@yahoo.co.uk (S.G.); milenagos@abv.bg (M.G.); angelova_ts@abv.bg (T.A.); 2 Institute for Roses and Aromatic Plants, Agricultural Academy, 49 Osvobojdenie Blvd., 6100 Kazanlak, Bulgaria; anadobreva@abv.bg; 3 The Stephan Angeloff Institute of Microbiology, Bulgarian Academy of Sciences, 26 Acad. G. Bonchev Str., 1113 Sofia, Bulgaria; milkamileva@gmail.com; 4 Department of Pharmacognosy, Faculty of Pharmacy, Medical University of Sofia, 2 Dunav Str., 1000 Sofia, Bulgaria; zlatina.kokanova@pharmfac.mu-sofia.bg (Z.K.-N.); pnedialkov@pharmfac.mu-sofia.bg (P.N.)

**Keywords:** rose subcritical water extracts, bioactive compounds, in vivo test systems, in vitro test system, cytotoxicity, genotoxicity, cytogenetic tests

## Abstract

Regulatory changes in the EU for safety purposes require strict control and high safety standards for essential oils obtained by steam distillation, as they are classified as chemical mixtures with potential toxic effects. Subcritical water extracts (SWEs) are considered safer. This study evaluated the cytotoxicity and genotoxicity of SWEs from *Rosa damascena* Mill. and *Rosa alba* L. in three test systems at different hierarchical levels: higher plants (root meristems of *Hordeum vulgare*), somatic cells of *Mus musculus* ICR strain, and human lymphocytes in vitro. The chromatographic fingerprint of the extracts revealed the presence of key components such as flavonoids, phenolic acids, and glycoside derivatives, with species-dependent variations and concentrations. No significant cytotoxicity was detected in the concentration range of 6–20%. SWE from *R. alba* showed a higher level of safety at high doses. Genotoxicity tests showed a weak, dose-dependent induction of chromosomal aberrations and micronuclei in barley and lymphocytes (greater in *R. alba*), a lack of genotoxicity in mouse bone marrow, and a slight increase in micronuclei in mouse erythrocytes after exposure to *R. alba* extract. The results highlight the suitability of SWEs from *R. damascena* and *R. alba* for safe application in the medical, food, and cosmetic industries.

## 1. Introduction

Bulgarian rose oil, obtained by the classical method of steam distillation, is considered a world standard of quality and unique aroma [1,2]. In Bulgaria, *Rosa damascena* Mill. (especially its subspecies “Kazanlak rose”) and *Rosa alba* L. are the main oil-producing species cultivated, due to the favorable climatic conditions in the Rose Valley near the city of Kazanlak [3,4]. As members of the Rosaceae family, despite their similarities, the two oil-bearing roses also have significant differences, especially in terms of aroma, chemical composition, and essential oil yield [5]. *Rosa damascena* yields a higher yield of essential oil, while *Rosa alba* oil, although more challenging to extract, is more expensive and has a lighter and more subtle aroma [5,6].

*R. damascena* Mill. is valued both as an ornamental plant and as a key raw material for the perfume industry, as well as for its pharmacological potential [7]. Numerous studies highlight the abundance of biologically active substances in rose essential oil. This phytochemical richness underpins the use of *R. damascena* in cosmetics, dermatology, and the food industry, with well-documented aromatic, antioxidant, and dermal-protective properties [8,9,10,11,12]. Recent studies highlight its redox-modulating, chemopreventive, and anticancer effects, which have been linked to polyphenols and monoterpenes [12,13]. Other studies report antiviral, antimicrobial, antioxidant, vasodilatory, anti-inflammatory, antiproliferative and cytoprotective effects associated with rose constituents [14,15,16,17,18,19,20,21]. Some studies exist about the cytotoxic and genotoxic activities [22] and the genoprotective effect of *R. damascena* hydrosol derived by water-steam distillation [23].

Rose oil, obtained through the water-steam distillation of fresh *R. alba* L. blossoms, is widely used in perfumery, aromatherapy, and dermatology worldwide [24]. Bulgarian *R. alba* L. essential oil has demonstrated well-expressed antiradical, antimicrobial, and antiviral effects [19,25]. Its hydrosol exhibits antioxidant activity [26] and may help prevent cardiovascular diseases [27]. Rose oil does not exhibit statistically significant cytotoxic or genotoxic activities in the plant test system. Cytogenetic analysis showed its low cytotoxic and genotoxic effects in human lymphocytes in a dose-dependent manner. It demonstrated a good defense potential against the experimental mutagen N-methyl-N’-nitro-N-nitrosoguanidine (MNNG) [28]. The biological properties of the technological products obtained by steam distillation of essential oils from *R. damascena* and *R. alba* are due to the presence of a wide range of bioactive substances.

*R. damascena* oil contains key monoterpene alcohols (geraniol, citronellol, nerol, linalool) and phenylethyl alcohol (present in both rose essential oil and absolute, which defines both its characteristic fragrance and its valuable biological properties [29,30,31]. Beyond volatile constituents, *R. damascena* contains flavonoids, tannins, glycosides, anthocyanins, terpenes, fatty oils, vitamin C, and organic acids, with over 95 components identified [32,33]. Chromatographic studies show that *R. alba* essential oil is dominated by oxygenated monoterpenes (geraniol, nerol, citronellol) and traces of eugenol/methyleugenol. These monoterpenes suppress lipid peroxidation, with citronellol most active [25]. Although often classified as a citronellol-type [5], *R. alba* oil exhibits compositional variability, ranging from citronellol-to geraniol-dominant profiles [34,35]. Industrial surveys report geraniol at approximately 16–34% and citronellol at approximately 7–29% in oils. *R. alba* hydrosols are rich in phenylethyl alcohol, and blossom headspace highlights phenylethyl alcohol, citronellol, and geraniol, with no methyleugenol detected [36]. Methyleugenol is generally absent or present only in trace amounts, in line with safety standards [37], and geraniol is a key pharmacologically active constituent [38].

In addition to their pharmacological relevance, rose oils’ composition and quality are shaped by distillation technology, genotype, environment, harvest stage, pruning, storage, and climate [3,30,39]. Extraction of rose oil and bioactives relies on various technologies.

Hydro-distillation and steam distillation are two conventional methods used by industries to obtain valuable essential oils. They are considered environmentally safe, but these methods are incredibly time-consuming. Other disadvantages include the loss of many volatile substances during these procedures, low extraction efficiency, degradation of some unsaturated compounds, and substantial energy consumption [40]. Solvent-based extraction methods (using hexane, ethanol, or chloroform) are widely used for rose’s oil and bioactives, but these approaches can leave residues and pose safety concerns, raising environmental and health issues [18,41,42]. In response to the limitations mentioned above, and to overcome the drawbacks, research has shifted toward new, greener extraction technologies. Among these, subcritical fluid or water extraction (SWE) stands out for offering high purity, reduced energy consumption, and minimal ecological impact [43,44,45,46,47]. Compared to conventional methods, it is faster, safer, and more selective, requiring minimal post-processing [48,49,50]. SWE’s effectiveness for essential oils, polyphenols, and environmental matrices has been well documented [47,51]. It was reported that these methods enhanced both the yield and quality of the essential oil [52] and were successfully applied for essential oil recovery from certain seeds [53] and wood [40]. SWE successfully extracts bioactive-rich antioxidant compounds from medicinal and aromatic plants (e.g., grape, beetroot, ginger, *Matricaria chamomilla* L.), consistently yielding superior phytochemical profiles and higher antioxidant activities [50,51,52,53,54,55,56,57,58,59,60].

It is water used while lack the residues of toxic solvents [61,62]. This is particularly relevant for rose-derived products across perfumery, cosmetics, pharmaceuticals, and medicine.

Although SWE has demonstrated promising results for certain medicinal plants and for *R. damascena*, research on *R. alba* and comprehensive safety profiling of rose SWEs remains limited, representing a key knowledge gap [6,46,48]. This context underscores the need for studies that not only address current regulatory requirements [63] for the validation of both the safety and biological activity of these products [64], but also fill the existing gap for their phytochemical characterization.

The aim of this study was to evaluate the chromatographic profile, cytotoxicity and genotoxicity of SWEs from *Rosa damascena* Mill. and *Rosa alba* L. in three test systems at different hierarchical levels: higher plants (root meristems of *Hordeum vulgare*), somatic cells of the *Mus musculus* ICR strain and human lymphocytes in vitro.

## 2. Results

### 2.1. Composition Data of SWEs from R. damascena Mill. and R. alba L.

The chemical composition of the subcritical water extracts is presented in Table 1.

UHPLC-HRMS/MS analysis revealed distinct phytochemical profiles in the SWEs of *Rosa damascena* and *Rosa alba*. Both rose SWEs contained a wide range of flavonoids, phenolic acids, and glycosidic derivatives, but their quantitative distribution varied. The major compound in both extracts was 2-phenylethanol O-hexoside, detected at markedly higher levels in *R. damascena* (112.94 µg/mL) compared to *R. alba* (60.58 µg/mL), with relative percentages of 21.97 and 14.92, respectively. Ellagic acid, a key antioxidant, was also abundant, with a nearly threefold higher concentration in *R. damascena* (60.86 µg/mL, with relative % 11.84) than in *R. alba* (21.88 µg/mL with relative % 5.39). In contrast, catechin and epicatechin were present in low amounts in *R. damascena* (0.27 and 0.11 µg/mL, respectively) but were significantly enriched in *R. alba* (5.53 with relative % 1.36 and 2.11 µg/mL with relative % 0.52). Kaempferol-3-O-glucoside represented one of the predominant flavonoids, with similar concentrations in *R. damascena* (83.57 µg/mL) and *R. alba* (82.12 µg/mL). Other kaempferol derivatives, including kaempferol-3-O-galactoside, kaempferol-3-O-xyloside, and kaempferol-3-O-arabinoside, were present in comparable amounts between the two species. Likewise, quercetin glycosides such as hyperoside and isoquercitrin showed similar levels in both rose extracts. However, quercetin was substantially higher in *R. damascena* (11.58 µg/mL with relative % 2.25) compared to *R. alba* (0.70 µg/mL with relative % 0.17).

Overall, *R. damascena* SWE was characterized by higher levels of phenylethyl derivatives, ellagic acid, and aglycone flavonols (quercetin, kaempferol), while *R. alba* shows notably higher quercetin and catechin-related content. These compositional differences highlight species-dependent variations in phenolic and volatile glycoside accumulation under SWE.

### 2.2. Cytotoxic Effects of Rose SWEs

#### 2.2.1. Mitotic Index (MI)

**Figure 1:**
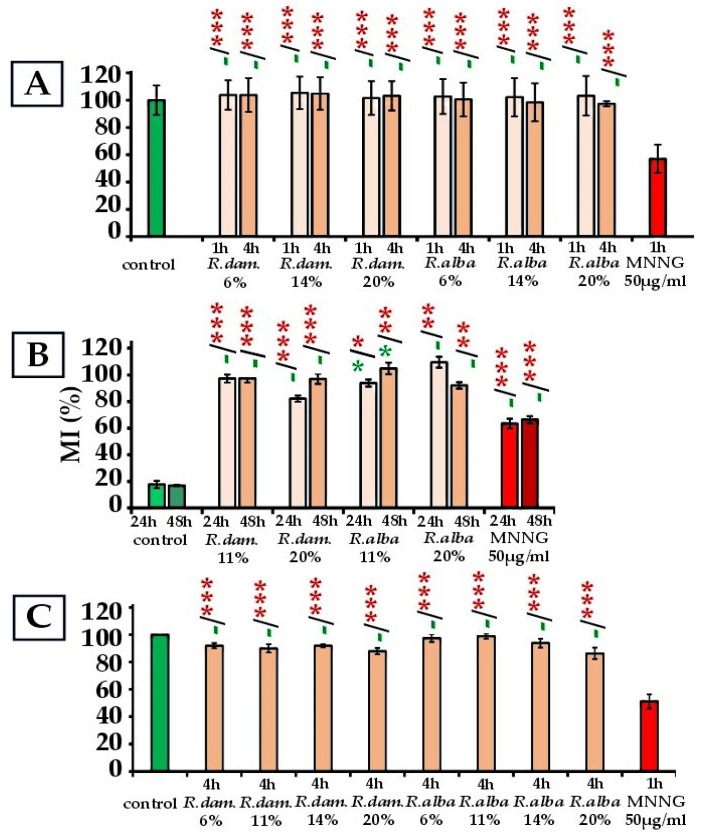
Cytotoxic activity of *R. damascena* and *R. alba* SWEs assessed by the value of MI in: *H. vulgare* (**A**); mouse bone marrow cells (**B**); human lymphocytes (**C**). Mitotic activity (MI) was evaluated as a percentage of the negative control. * *p* < 0.05, ** *p* < 0.01, *** *p* < 0.001, and (-) non-significant versus negative control (before the slash), versus positive control MNNG (after the slash). Green—comparison to the negative control; red—comparison to the positive control. This facilitates interpretation.

*R. damascena* subcritical water extract, applied alone to barley (*Hordeum vulgare*) for 1 or 4 h at concentrations of 6–20%, yielded MI values of 101.61% ± 12.37 to 105.4% ± 11.83 relative to the control, with no significant differences observed (*p* > 0.05) (Figure 1A). In human lymphocytes (4 h exposure, 6–20%), most MI values remained within ≈90–92% of control, with the lowest value being 88.00% ± 2.22 at 20% and still non-significant (*p* > 0.05) (Figure 1C). Bone marrow cells showed values ranging from 97.29% ± 3.06 (11%) to 82.18% ± 2.33 (20%/24 h), with a significant reduction evident only at 20%/24 h (*p* < 0.05) (Figure 1B). Overall, across barley and lymphocytes, no cytotoxicity was detected, even at 20%. In human lymphocytes, reductions in MI were minor (≤12%) and statistically non-significant.

Likewise, mouse cells exposed to 11% or 20% extract for 24 or 48 h did not exhibit suppression of mitotic activity (*p* > 0.05), aside from 20%/24 h, which showed a small, transient decline/reduction (*p* < 0.05) that returned to control-range by 48 h (97.07% ± 3.86, ≈3% below control; *p* > 0.05).

In contrast, the positive control MNNG (50 μg/mL) caused pronounced cytotoxicity, reducing MI to 56.99% ± 10.22 in barley (*p* < 0.001), 63.45% ± 3.25 to 66.41% ± 2.68 in ICR mice (*p* < 0.05; *p* < 0.01), and 51.20% ± 5.20 in lymphocytes (*p* < 0.001).

In summary, across plant, murine, and human systems, *R. damascena* subcritical water extract appears non-cytotoxic up to 20%, with the only apparent effect being a mild, transient decrease in MI at 20% after 24 h in mouse bone marrow (*p* < 0.05). All rose SWEs groups differed significantly from the MNNG group.

The *R. alba* SWE underwent the same treatment schemes, and the data are presented in Figure 1.

In *H. vulgare*, MI values across treatments (1 h and 4 h) remained close to the control (97.31% ± 11.83 at 20%/4 h–103.23% ± 14.52 at 20%/1 h) (*p* > 0.05), indicating the absence of cytotoxic effects. Similarly, bone marrow cells of ICR mice treated with 11% or 20% *R. alba* SWEs for 24 or 48 h displayed MI values within the control range (92.16% ± 2.44 at 20%/48 h–109.48% ± 4.08 at 20%/24 h; *p* > 0.05). In human lymphocytes (4 h, 6–20%), MI values ranged from 86.40% ± 4.21 (20%) to 99.00% ± 2.00 (11%), with no significant differences from controls (*p* > 0.05). Even the lowest value (86.4% at 20%) was indistinguishable from the control.

Overall, *R. alba* SWEs caused no cytotoxicity in any of the test systems at concentrations up to 20%. Furthermore, all treatment groups differed from MNNG (*p* < 0.001) (Figure 1).

The comparative analysis of the subcritical water extracts of *R. damascena* and *R. alba* revealed that both extracts were non-cytotoxic across the three experimental test systems employed. In barley root meristems, neither extract reduced MI when administered alone, and both demonstrated the capacity to counteract the cytotoxic effect of MNNG, with *R. damascena* showing slightly stronger recovery of mitotic activity. In mammalian systems, *R. alba* SWEs showed no cytotoxicity at concentrations up to 20% at any exposure time, while *R. damascena* caused a mild but statistically evident reduction in mitotic activity at 20% after 24 h, which resolved by 48 h. In human lymphocytes, both extracts were non-cytotoxic up to 20%, with only minor, non-significant decreases in mitotic activity at the highest concentrations. Taken together, these findings suggest that *R. alba* may be considered marginally safer at higher concentrations, especially in mammalian cells.

#### 2.2.2. PCE/(PCE + NCE) Ratio

**Figure 2:**
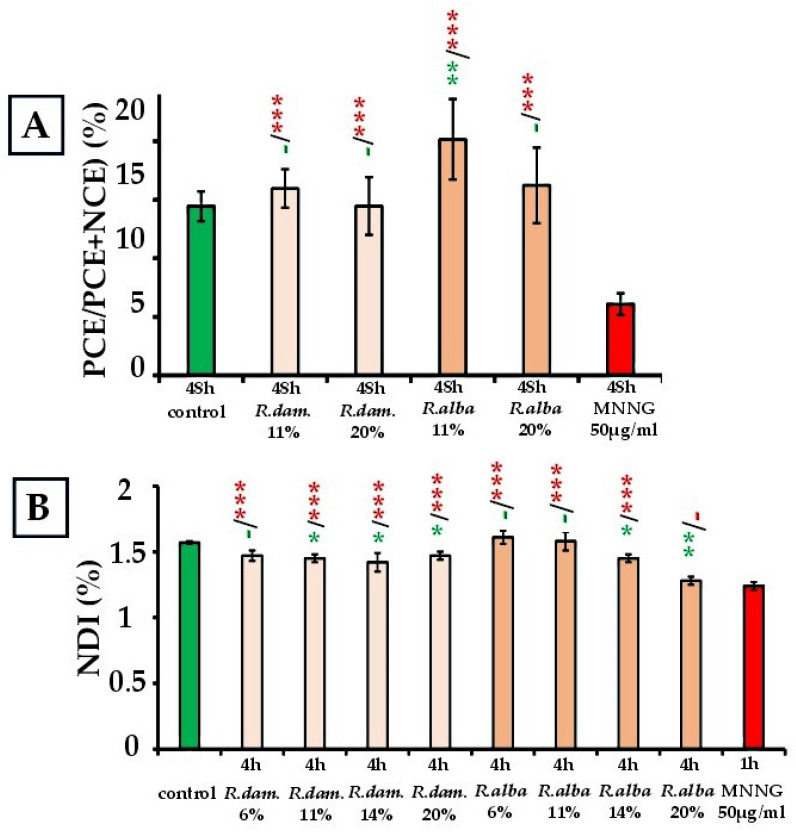
Cytotoxic activity of *R. damascena* and *R. alba* SWEs, assessed by PCE/(PCE + NCE) ratio in mouse peripheral blood erythrocytes (**A**) and NDI in human lymphocytes (**B**). * *p* < 0.05, ** *p* < 0.01, *** *p* < 0.001, and (-) non-significant versus negative control (before the slash), versus positive control MNNG (after the slash). Green—comparison to the negative control; red—comparison to the positive control. This facilitates interpretation.

As for the in vivo mammalian erythrocyte micronucleus test, the ratio of polychromatic erythrocytes (PCE) to total erythrocytes (PCE + NCE) was determined for each animal and used as a biomarker of cytotoxicity (Figure 2A).

For *R. damascena* SWE (15.95% ± 1.65/11% and 14.46% ± 2.46/20%), values showed no dose-dependent trend and remained close to the negative control (14.44% ± 1.27; *p* > 0.05), but were elevated compared with the positive control MNNG (6.09% ± 0.93; *p* < 0.001).

Treatment with *R. alba* SWEs, on the other hand, displayed a dose-related difference between the two doses tested: the 20% dose yielded 16.23% ± 3.22, while the 11% dose produced 20.16% ± 3.44; the 11% value was statistically higher than both the control (*p* < 0.01) and the 20% dose (*p* < 0.05), whereas the 20% value was indistinguishable from control (*p* > 0.05).

By contrast, MNNG treatment caused marked suppression of PCE formation (6.09% ± 0.93; *p* < 0.001), confirming strong cytotoxicity and validating the assay’s sensitivity. Overall, neither *R. damascena* nor *R. alba* induced suppression of erythropoiesis; increases in the PCE/(PCE + NCE) ratio at some doses indicate preserved erythroid proliferation relative to the mutagenic control (some doses even maintained PCE proportions above the mutagenic control).

#### 2.2.3. Nuclear Division Index (NDI)

Exposure of human lymphocyte cells to *R. damascena* SWE at increasing concentrations (6%, 11%, 14%, 20%), yielded values ranging from 1.47% ± 0.04 to 1.42% ± 0.07, with lower indices than control at 11%, 14%, and 20% (control: 1.57% ± 0.01; *p* < 0.05), whereas the lowest dose (6%; 1.47% ± 0.04) did not differ from control (no reduction in proliferation was detected vs. negative control) (*p* > 0.05) (Figure 2B). *R. alba* SWE showed similar trends: NDI remained comparable to the control value at low and moderate concentrations (6%, 11%, 14%) (1.61% ± 0.05 to 1.58% ± 0.07), while the highest concentration (20%) decreased NDI to 1.28% ± 0.03, a value not different from MNNG (1.24% ± 0.03; *p* > 0.05), indicating suppressed cell division activity. These data suggest a differential impact of the two extracts: *R. damascena* appears to exert mild, dose-dependent suppression of lymphocyte proliferation, whereas *R. alba* is largely non-cytotoxic at low to moderate doses but exhibits some cytotoxic activity at higher concentrations (14% and 20%).

### 2.3. Genotoxic Effects of Rose SWEs

#### 2.3.1. Induction of Chromosome Aberrations

**Figure 3:**
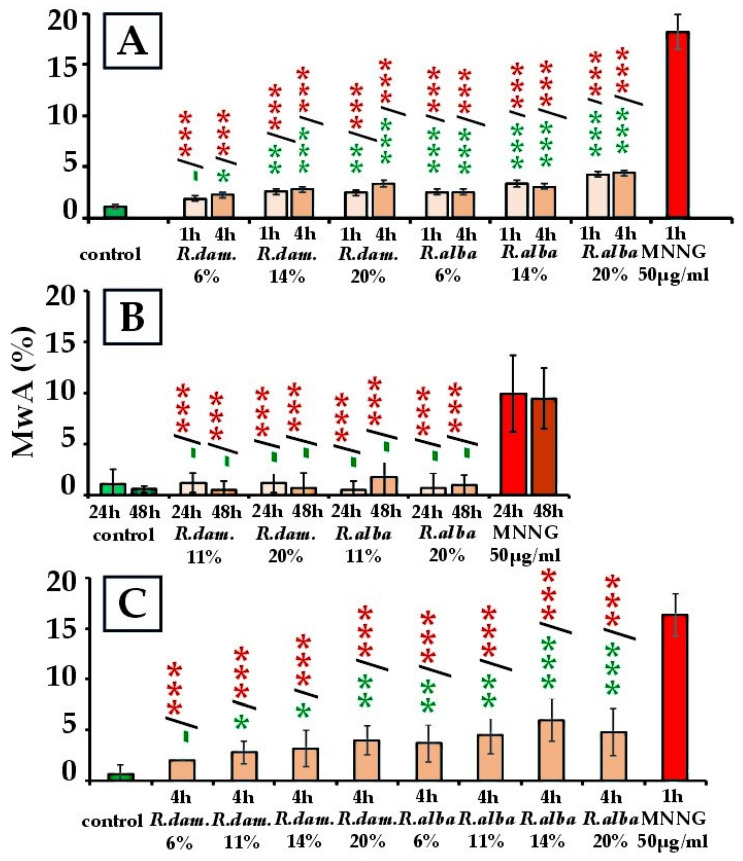
Genotoxicity results of *R. damascena* and *R. alba* SWEs, assessed by the frequency of metaphases with chromosome aberrations (MwA) in *H. vulgare* (**A**), mouse bone marrow cells (**B**), and human lymphocytes (**C**). The statistical significance is denoted by * *p* < 0.05, ** *p* < 0.01, *** *p* < 0.001, and (-) non-significant versus negative control (before the slash) versus positive control MNNG (after the slash). Green—comparison to the negative control; red—comparison to the positive control. This facilitates interpretation.

The genotoxic effects of *R. damascena* and *R. alba* SWEs were evaluated by analyzing the frequency of metaphases with chromosome aberrations (MwA) across three test systems (Figure 3).

In barley and human lymphocytes, *R. damascena* SWE produced modest but clear elevations in chromosomal aberrations (CA). In lymphocytes, frequencies rose from 2.00% ± 0.0 (6%; *p* < 0.05 vs. control) to 4.00% ± 1.40 (20%; *p* < 0.01 vs. control). In barley, values ranged from 1.92% ± 0.28 (6%; *p* < 0.05) to 3.40% ± 0.29 (20%; *p* < 0.001), compared with 1.13% ± 0.18 in controls. In ICR mouse bone marrow cells, no significant differences from control were detected at either 24 h or 48 h (*p* > 0.05). Aberration frequencies ranged from 1.25% ± 0.97 (20%, 24 h) to 0.5% ± 0.87 (11%, 48 h), close to that of the controls.

All *R. alba* SWE treatments in barley and human lymphocytes yielded higher aberration frequencies than the respective controls (Figure 3A,C). In barley, *R. alba* SWE raised CA frequencies with an overall concentration-related pattern, with the highest effect at 20% extract (4.40% ± 0.23; *p* < 0.001) after four hours of exposure. In human lymphocytes, the extract induced a dose-related increase in chromosomal aberrations, with the maximum at 14% (6.0% ± 2.1; *p* < 0.001). In bone marrow cells, no significant increases were observed at either 24 or 48 h, with frequencies ranging between 0.50% ± 0.87 (11%, 24 h) and 1.75% ± 1.85 (11%, 48 h), close to that of the controls (1.14% ± 1.46 at 24 h; 0.6% ± 0.3 at 48 h; *p* > 0.05) (Figure 3B).

The clastogenic activity of both extracts at all studied concentrations was far lower (*p* < 0.001) than that of the potent mutagen MNNG (50 μg/mL) in all three test systems (18.22% ± 1.72 in *H. vulgare*; 10.0% ± 3.74 in bone marrow cells at 24 h and 9.50% ± 2.96 at 48 h; 16.30% ± 2.10 in human lymphocytes) (Figure 3). As expected, MNNG markedly increased the number of damaged cells.

Overall, both *R. damascena* and *R. alba* SWEs elicited weak, statistically supported concentration-related increases in chromosomal aberrations in *H. vulgare* and human lymphocytes, with the largest responses observed in lymphocytes; at comparable concentrations, *R. alba* generally caused slightly higher aberration frequencies than *R. damascena*, peaking at 14% (6.0% ± 2.1), consistent with a slightly higher burden or potency of genotoxic constituents under subcritical extraction conditions. In contrast, neither extract produced detectable genotoxic effects in vivo in mouse bone marrow cells at 24 or 48 h, within the tested conditions. Taken together, these findings support that *R. alba* exhibits greater in vitro genotoxic potential than *R. damascena*, while both species remain much less damaging than MNNG, likely reflecting species-specific differences in phytochemical composition.

**Figure 4:**
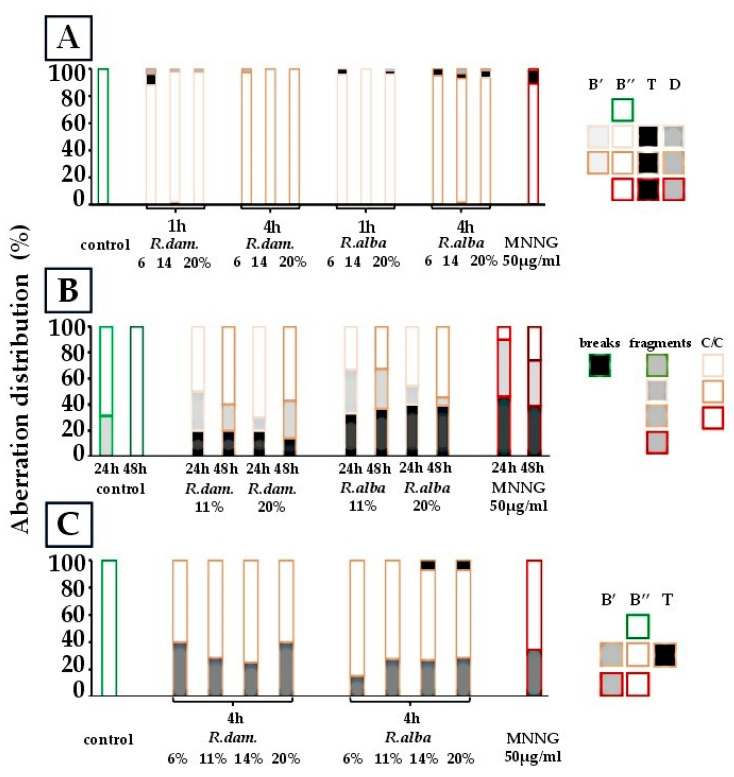
Distribution of aberrations observed in genotoxicity analyses after treatment with rose SWEs in *H. vulgare* (**A**), mouse bone marrow cells (**B**), and human lymphocytes (**C**).

The distribution of CA observed after treatment with the same rose SWEs in *H. vulgare*, mouse bone marrow cells, and human lymphocytes is presented in Figure 4.

Analysis of the CA distribution revealed that treating barley with *R. damascena* extract mainly resulted in isochromatid breaks (B’’), with percentages ranging from 88.0% to 100% across all concentrations and exposure times (Figure 4A). Small amounts of chromatid breaks (B’) were only observed at 14%/1 h (2.22%). Translocations (T) were rare, observed only at 6%/1 h (8.0%), and deletions (D) appeared at very low levels (2.22–2.56%) in some groups. At 14% and 20%/4 h, aberrations consisted solely of B’’ (100%).

In vivo treatment of ICR mouse bone marrow with *R. damascena* SWEs induced low levels of chromosomal aberrations (Figure 4B). At 11% extract (24 h), 10 aberrations were observed (2.5/100 metaphases), whereas 20% extract yielded 11 aberrations (2.75/100 metaphases), predominantly centromere–centromere fusions (63.6%), with one telomere–telomere fusion. Frequencies declined at 48 h (1.25–1.75/100 metaphases). Overall, centromere–centromere fusions were the most frequent (50–64%), while chromatid-type breaks and fragments were less common.

In lymphocyte cultures (Figure 4C), *R. damascena* SWE primarily induced isochromatid breaks (B’’) (60.0–75.9%) at concentrations of 6–20%, with chromatid breaks (B’) accounting for 25.0–40.0%. No translocations were observed.

*R. alba* SWE in barley produced distributions likewise dominated by B’’ (Figure 4A). After 1 h extract treatment, B’’ accounted for 95.8–100%, with translocations (2.53–4.17%) and deletions (1.27%) observed at some concentrations. After 4 h, B’’ remained high (91.23–95.24%), while translocations (3.51–5.13%) and deletions (1.28–3.51%) were more frequent. Notably, 14% (1 h) yielded 100% isochromatid breaks, while after 4 h of treatment, aberration types diversified, although B’’ remained most common.

In ICR mice (Figure 4B), *R. alba* SWE caused minimal chromosomal damage: at 11%, 24 h exposure resulted in 4 events (1.0/100 metaphases); at 48 h, this rose to 9 events (2.25/100). At 20%, 7 aberrations occurred at 24 h (1.75/100), and 9 at 48 h (2.25/100). Centromere-centromere fusions (c/c) were predominant, with chromatid breaks becoming more apparent at higher doses and longer exposures. In human lymphocytes (Figure 4C), *R. alba* SWE mainly induced B’’ (64.3–84.6%) and B’ (15.4–28.6%). Unlike *R. damascena*, it also yielded low but consistent levels of translocations (≈7.0–7.1%) at 14–20%.

**Figure 5:**
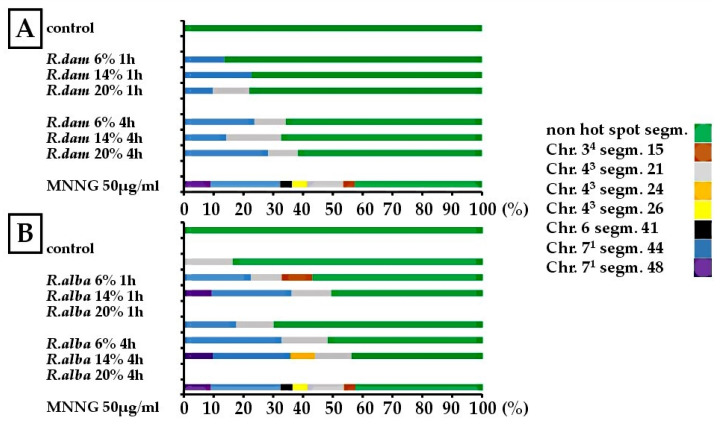
Aberration “hot spots” observed in the reconstructed* karyotype MK14/2034 of *H. vulgare* after treatment with three different concentrations of *R. damascena* (**A**) and *R. alba* (**B**) SWEs. * The karyotype of *H. vulgare* was reconstructed by combining two simple reciprocal translocations: one between parts of chromosomes 1 and 7, and the other between parts of chromosomes 3 and 4.

Identification of chromosomal “hot spots” in the reconstructed barley karyotype MK 14/2034 can provide further insights into how subcritical rose extracts affect *H. vulgare* root meristem cells (Figure 5).

In the control variants, no hot spot regions were observed. After 1 h of *R. damascena* treatments, four hot spots were identified (Figure 5A). Recurrent involvement of chromosome 7^1^ segment 44 was seen across all concentrations (13.6% at 6%, 22.7% at 14%, 8.9% at 20%). At 20%, chromosome 4^3^ segment 21 was also affected (12.2%). Following a 4-h exposure, the number of hot spots increased, with segment 21 of chromosome 4^3^ (10.5% at 6%, 18.4% at 14%, and 10% at 20%) and segment 44 of chromosome 7^1^ affected across all concentrations. However, most chromosome regions showed no hot spots (61.7–67.3%).

The positive control MNNG (50 μg/mL) exhibited the broadest hot spot profile, causing six hot spots across the 48 chromosomal segments examined. These aberrations were found in the following segments: segment 15 of chromosome 3^4^ (3.9%); chromosome 4^3^, segments 21 (12.2%) and 26 (5.0%); segment 41 of chromosome 6 (3.9%); and segments 44 (23.4%) and 48 (9.0%) of chromosome 7^1^. Overall, these hot spots represented 57.4% of the total damage, emphasizing the mutagen’s preference for fragile chromosomal sites.

The effects of *R. alba* SWE (Figure 5B) indicated that after 1 h of treatment, 50.7–83.7% of all chromosome segments were free of aberration hot spots. Three hot spots were identified: chromosome 4^3^ segment 21 (16.3% at 6%; 10.3% at 14%; 13.3% at 20%), chromosome 3^4^ segment 15 (10.3% at 14%), and chromosome 7^1^ segments 44 (22.4–26.7% at 14–20%) and 48 (9.3% at 20%). The 4-h *R. alba* SWE exposure led to an increase in hot spots to four. At 6%, aberrations were found in chromosome 4^3^ segment 21 (12.5%), chromosome 7^1^ segment 44 (17.5%), and regions without hot spots (70%). At 14%, chromosome 4^3^ segment 21 (15.4%) and chromosome 7^1^ segment 44 (32.7%) were affected, while at 20%, chromosome 4^3^ segment 24 (8.2%) and chromosome 7^1^ segment 44 (26%) dominated. Across all concentrations, non-hot spot regions accounted for 51.9–70%.

#### 2.3.2. Induction of Micronuclei

**Figure 6:**
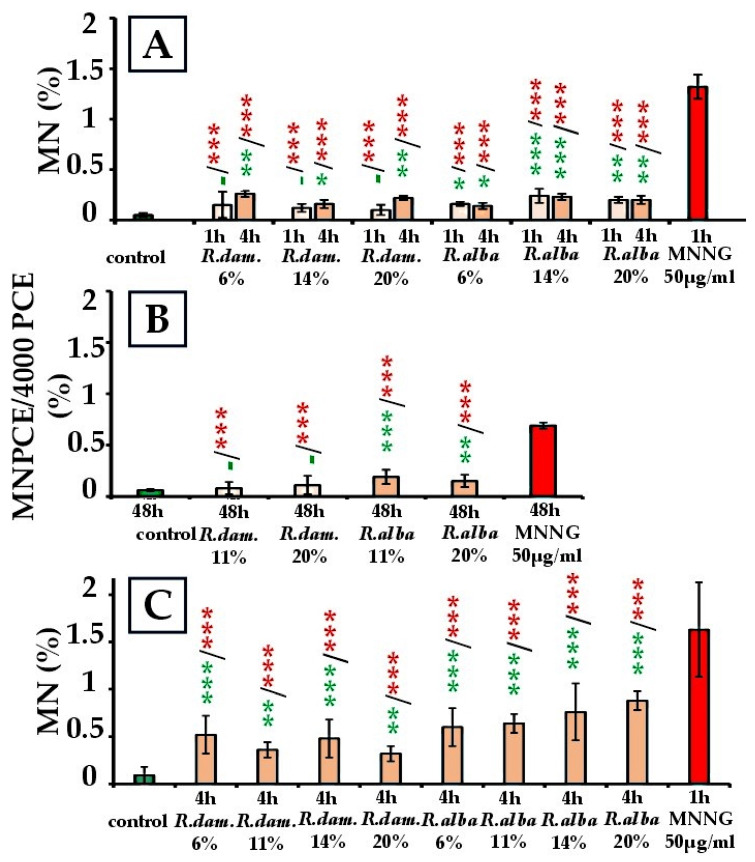
Genotoxic effect of *R. damascena* and *R. alba* SWEs, assessed by the frequency of induced MN in: *H. vulgare* (**A**), mouse peripheral blood erythrocytes (**B**), and human lymphocytes (**C**). * *p* < 0.05, ** *p* < 0.01, *** *p* < 0.001, and (-) non-significant versus negative control (before the slash) versus positive control MNNG (after the slash). Green—comparison to the negative control; red—comparison to the positive control. This facilitates interpretation.

A statistically significant genotoxic effect was observed in both *R. damascena* and *R. alba* SWEs, depending on the test system (Figure 6).

In *H. vulgare*, a 1-h treatment with *R. damascena* SWE resulted in MN frequencies ranging from 0.15% ± 0.13 (6%) to 0.10% ± 0.05 (20%), with no significant difference from the control (*p* > 0.05). Following 4 h of treatment, MN frequencies increased significantly to 0.26% ± 0.03 (6%; *p* < 0.01), 0.16% ± 0.04 (14%; *p* < 0.05), and 0.22% ± 0.02 (20%; *p* < 0.01) compared to the control (0.05% ± 0.02) (Figure 6A). In mouse peripheral blood erythrocytes, 48 h of treatment yielded values of 0.11% ± 0.09 (20%) and 0.08% ± 0.06 (11%), which were comparable to the control (0.06% ± 0.01; *p* > 0.05), indicating no in vivo genotoxicity (Figure 6B). Conversely, in human lymphocytes, MN induction increased at all concentrations after 4 h: 0.52% ± 0.20 (6%), 0.36% ± 0.08 (11%), 0.48% ± 0.20 (14%), and 0.32% ± 0.08 (20%) versus control (0.09% ± 0.09) (*p* < 0.01–0.001) (Figure 6C). Although these values increased, they remained below MNNG levels (1.63% ± 0.50) (*p* < 0.001). In both plant and mouse systems, the MN frequencies remained far below their MNNG references (1.32% ± 0.12 for *H. vulgare* and 0.69% ± 0.03 for mouse erythrocytes; *p* < 0.001).

For *R. alba SWEs*, a similar overall pattern was observed. In *H. vulgare*, MN after 1 h reached 0.16% ± 0.02 (6%), 0.24% ± 0.07 (14%), and 0.20% ± 0.03 (20%), while after 4 h, frequencies were 0.14% ± 0.03, 0.23% ± 0.03, and 0.20% ± 0.04, each above the control (*p* < 0.05–0.001) (Figure 6A). In mouse peripheral blood erythrocytes, unlike *R. damascena*, *R. alba* SWE elevated MN relative to the negative control: 0.15% ± 0.06 (20%; *p* < 0.01) and 0.19% ± 0.07 (11%; *p* < 0.001) (Figure 6B). In human lymphocyte cultures, *R. alba* SWE produced a genotoxic effect across doses after 4 h, ranging from 0.60% ± 0.20 (6%) to 0.88% ± 0.10 (20%), all above control (*p* < 0.001) (Figure 6C). Nonetheless, in all systems, MNNG produced larger responses (*p* < 0.001). Overall, both *R. damascena* and *R. alba* SWEs induced moderate yet significant MN induction in *H. vulgare* and human lymphocytes. However, only *R. alba* SWE showed a clear in vivo genotoxicity in mammalian cells.

The most pronounced responses were observed in lymphocytes, especially after exposure to *R. alba* SWE, suggesting a higher genotoxic potential than *R. damascena*.

## 3. Discussion

In the present work, we investigated the phytochemical profile of SWEs from the flowers of Bulgarian oil seeds *R. damascena* Mill. and *R. alba* L. using UHPLC-HRMS/MS analysis and evaluated their potential cytotoxic and genotoxic safety in experimental models in vitro and in vivo. We employed three different test systems: plant root meristem cells, somatic cells of the *Mus musculus* ICR strain, and human lymphocyte cultures, using genotoxicity tests. The phytochemical profile of the SWEs helped us in interpreting their biological properties.

Subcritical water extraction is an important alternative to steam distillation of essential oils. The advantage of this method is that other valuable components that are not contained in essential oils are also included in the extract, such as some bioflavonoids in their glycoside and aglycone forms [65], polyphenols and procyanidins [66], organic acids [67], carbohydrates [68], alkaloids [69], terpenes [70], etc. The uniqueness of the method is as follows: (i) water is non-toxic and is more suitable for the extraction of medicinal plants, as the extracts can be safely consumed by humans or animals; (ii) the polarity of water molecules can dramatically decrease with increasing temperature and dissolve less polar molecules. Here, the principle of “like dissolves like” is followed [51].

As shown in Table 1, the main components belong to the aforementioned classes of chemical compounds; however, they are present in varying abundances in the two extracts. When comparing the chromatographic profiles of the essential oils from the two roses obtained by steam distillation, significant differences were observed [18]. *R. damascena* oil is characterized by a high content of terpene alcohols, such as citronellol, geraniol, nerol and linalool, as well as hydrocarbons with chain lengths of C17, C19 or C21. *Rosa alba* essential oil contains the same lipophilic components, but in different amounts [18].

As a rule, the biological properties of each phytocomplex are closely related to its chemical composition. In order to conduct further studies on the pharmacological potential of BHCs from *Rosa alba* and *Rosa damascena*, in accordance with the current regulatory requirements of the European Parliament and the Council of the European Union, 2008, and to validate their toxicological safety, in this work, we performed a screening of their cytotoxic and genotoxic activities.

### 3.1. Cytotoxic Effects of Rose SWEs

Our comparative assessment of SWEs from *R. damascena* and *R. alba* confirms that both are mainly non-cytotoxic up to 14% (*v*/*v*) across all used in the study test systems, with only minor deviations at 20% (*v*/*v*) that do not indicate sustained adverse effects. Notably, the *R. damascena* extract caused a mild, transient decrease in the MI in mouse bone marrow at 20% after 24 h—an effect that resolved by 48 h, whereas *R. alba* showed no such effect even at the highest concentration tested. These findings align with recent research on related rose-based products obtained by water-steam distillation reporting minimal inherent cytotoxicity of hydrosols and distillation wastewaters [23,71,72]. The PCE/(PCE + NCE) ratio—an in vivo cytokinetic indicator of erythropoietic suppression in the micronucleus assay—remained comparable to or slightly higher than controls for both SWEs, in stark contrast to the marked reduction caused by MNNG. In *R. alba* at 11% (*v*/*v*), the slight increase in the PCE ratio may reflect a relative protective or adaptive effect on erythropoiesis, consistent with reported cytoprotective and antioxidant properties of rose products and constituents [23,28,73]. NDI revealed a sensitivity of proliferating human lymphocytes. The present study with SWEs shows a dose-dependent suppression, more pronounced for *R. damascena* extract. This difference between the two tested extracts corresponds with their phytochemical profiles. Our UHPLC-HRMS/MS results revealed dramatically higher concentrations of 2-phenylethanol O-hexoside (112.94 µg/mL) and ellagic acid (60.86 µg/mL) in *R. damascena* compared to *R. alba*, compounds recognized as modulators of cell cycling and antioxidant response [74,75,76,77]. At high concentrations, these compounds, along with increased levels of the aglycone flavonols quercetin (*R. damascena* contained 11.58 µg/mL versus 0.70 µg/mL in *R. alba*), can induce cytotoxic and pro-oxidant effects [78,79]. Conversely, *R. alba* SWE was enriched in catechin and epicatechin, flavan-3-ols linked to antioxidant and cytoprotective activity [80,81].

Although our study uses a subcritical water extract rather than an essential oil obtained by water steam distillation, the moderate cytotoxic features of *R. damascena* confirmed here in human lymphocytes and, to a lesser extent, mouse bone marrow—align with reports that essential oil or solvent extracts of *R. damascena* at higher doses induce oxidative stress mediated cell cycle arrest, apoptosis, and necrosis in mammalian cells [82,83]. Additionally, selective cytotoxicity of *R. damascena* towards malignant cell lines has been documented—showing concentration and time dependent effects on cancer cells with relative sparing of normal lymphocytes—supporting dose and cell type- dependent modulation by Rosa products [19,84,85,86,87,88,89]. The dual behavior of rose products is consistent with reports of biphasic actions of rose phytochemicals—protective at low levels but inhibitory to proliferation at higher ones [22,90]—and with the widely described hormesis of plant polyphenols and flavonoids, providing a mechanistic basis for the observed dose dependence [91,92,93,94]. Erythropoiesis in in vivo animal test systems appears largely unaffected by rose SWEs, whereas proliferating lymphocytes in vitro demonstrate sensitivity at higher extract concentrations. This differential outcome underscores the importance of employing multiple cytotoxicity endpoints across various test systems for comprehensive assessment. It suggests that the biological effects of these extracts predominantly target rapidly dividing cell populations (in vitro) rather than causing widespread cytotoxic damage, consistent with prior hydrosol data, which identified human lymphocytes as the most sensitive system [23,72].

### 3.2. Genotoxic Effects of Rose SWEs

The SWEs of *R. damascena* and *R. alba* caused low to moderate genotoxic effects in both plant (*H. vulgare*) and human lymphocyte test systems, but no genotoxicity was observed in mouse bone marrow. These findings correspond with our previous research on related rose-based products obtained by water-steam distillation [23,71,72]. In the present study, *R. alba* proved more genotoxic across the used endpoints than *R. damascena* at equivalent concentrations. This trend may be linked to the phytochemical composition of the tested rose SWEs. It is known that SWE can enhance recovery of flavonoids [62,65,95] and other less-volatile constituents, as demonstrated by the efficient recovery of hydrophilic polyphenols from *Rosa canina* fruits using pressurized hot water [96]. By contrast, liquefied-gas extraction with R134a targets more lipophilic/volatile fractions [97]. The intensity of SWE conditions (temperature and time) greatly influences extract composition: phenolic content and flavonoids generally increase with moderate heating up to an optimal point, then decrease due to thermal degradation and by-product formation [59,95,98] (trend magnitude depends on the compound). Similar extraction-dependent compositional shifts have been observed in non-rose species, where SWE enhances the recovery of bioactive fractions while maintaining cytogenetic safety, as seen in *T. ammi* essential oils [53] and in *A. malaccensis* oils [40]. These parallels strengthen our observation that *R. damascena* and *R. alba* SWEs exhibit low inherent genotoxicity. Since polyphenols and rose-derived monoterpenes are well-known for showing dose-dependent shifts between antioxidant/protective and pro-oxidant/genotoxic or cytotoxic behaviors [91,92,93], the cytogenetic responses observed likely reflect both the administered dose and extraction-related changes in composition.

*R. alba* SW extract contained higher catechin and epicatechin, flavonoid compounds that under some oxidative conditions can undergo redox cycling, whereas *R. damascena* SWE contained a much greater amount of ellagic acid, quercetin and kaempferol associated with DNA repair and antioxidant defense [93,99,100,101,102,103,104,105,106]. The dose-dependent induction of MN and CA responses observed here is consistent with hormetic patterns described for flavonoids, where low exposures boost antioxidant defenses whilst higher ones increase ROS and DNA damage [94]

Overall, differences in MN/CA induction and the inhibition of cell proliferation between *R. damascena* and *R. alba* can therefore be attributed not only to intrinsic species-related phytochemistry and administered dose but also to SWE-driven compositional shifts, emphasizing the need to correlate cytogenetic endpoints with phenolic/flavonoid/monoterpene content [95].

Both rose SWEs exhibited weak clastogenic activity compared to the direct mutagen MNNG. In barley, *R. damascena* SWE mainly caused B’’ aberrations with few exchange-type alterations, while *R. alba* produced a broader range, such as translocations and deletions, especially after a longer period of exposure. In bone marrow cells, both rose SWEs resulted in few aberrations, mostly centromere-centromeric fusions; *R. damascena* showed more consistent c/c dominance, whereas *R. alba* had slightly higher breakage rates at later time points. In human lymphocytes, *R. damascena* primarily induced B’’, while *R. alba* also caused low but consistent translocations.

Human lymphocytes proved to be the most sensitive test system, showing higher MN induction after exposure to both rose SWEs compared with the plant test system and ICR albino mice, especially *R. alba*, although still below MNNG levels. This heightened sensitivity may be linked to the enrichment of aglycones such as quercetin in *R. damascena* and catechins in *R. alba*, which modulate ROS and DNA repair differently [101,103,104,105,106,107,108]. This trend aligns with previous findings indicating that in vitro lymphocytes often detect subtle genotoxic effects not evident in more complex in vivo models [71,88,109,110,111,112]. Differences between plant, in vivo mammalian, and in vitro lymphocyte outcomes may also reflect variations in polyphenol pharmacokinetics and metabolism—the factors that determine the effective intracellular dose—an issue highlighted for polyphenols such as resveratrol and related compounds, and consistent with the lower in vivo findings reported here [113]. In *H. vulgare*, both extracts caused modest but significant increases in MN, consistent with earlier studies on rose hydrosol [23,71]. In mouse peripheral blood erythrocytes, only *R. alba* caused a moderate but significant increase in MN levels, whereas *R. damascena* did not—highlighting species- or extract-specific differences and potential in vivo relevance as was also observed in our studies using water steam distillation [23,71].

Our data generally support the evidence that the tested SWE rose extracts pose a low cytotoxic and genotoxic risk within a concentration range of 6–20% *v*/*v*, and the responses depend on the test system used.

## 4. Materials and Methods

### 4.1. Plant Material

Fresh flowers of *R. damascena* Mill. and *R. alba* L. were used as a raw material. They were picked up early in the morning (8.00–10.00 a.m.) [114] during flowering period of 2023. The plants are situated in the experimental field of the Institute for Roses and Aromatic Plants (IRAP), Kazanlak, Bulgaria.

### 4.2. Technology of SWE

The flowers were processed using a pressurized hot water automated system with a working volume of 2 L for the extractor. The apparatus and treatment conditions are provided in Dobreva et al. [6]. The extracts obtained were filtered and stored properly in dark, sealed flasks in a refrigerator.

### 4.3. UHPLC-HRMS Profiling and Semi-Quantification Determination of Phytochemicals

The UHPLC-HRMS profiling and semi-quantification of the main phytochemicals in the SWE extracts from *R. damascena* and *R. alba* were performed on a Dionex Ultimate 3000 RSLC system (Thermo Scientific, Germering, Germany), equipped with an SRD-3600 six-channel degasser, an HPG-3400RS high-pressure gradient pump, a WPS-3000TRS autosampler, and a TCC-3000RS column compartment, coupled to a Q Exactive Plus mass spectrometer (Thermo Scientific, Bremen, Germany). UHPLC separation was carried out on a Kromasil Eternity XT C18 column (2.1 × 100 mm, 1.8 μm; AkzoNobel, Angered, Sweden), equipped with a SecurityGuard ULTRA UHPLC EVO C18 pre-column (Phenomenex, Torrance, CA, USA), maintained at 40 °C. Chromatographic and mass spectrometric conditions were as described by Georgieva et al. [12].

Briefly, each chromatographic run was performed with a binary mobile phase consisting of water containing 0.1% (*v*/*v*) formic acid (A) and acetonitrile containing 0.1% (*v*/*v*) formic acid (B). A gradient program was used as follows: 0–1 min, held 5% B; from 1 to 25 min, the gradient was linearly chanced from 5 to 30% B; from 25 to 30 min, linearly changed from 30 to 40% B; from 30 to 32.5 min, linearly chanced from 40 to 95% B; finally, from 32.5 to 34.5 min, held 95% B. The flow rate was 0.3 mL min^−1^ and the sample injection volume was 2 µL. The system was conditioned for 4.5 min before injection. The operating conditions for the HESI source used in negative ionization mode were as follows: −2.5 kV spray voltage, 320 °C capillary and probe heater temperatures, sheath gas flow rate of 38 a.u., and auxiliary gas flow rate of 12 a.u. (a.u. refers to arbitrary values set by the Exactive Tune software, ver. 2.8 SP1), and S-Lens RF level of 50.00. Nitrogen was used for sample nebulization and as the collision gas in HCD cells. For metabolite identification purposes, the Full scan/ddMS2 (Top5) experiment was used, where in full MS mode the resolution, automatic gain control (AGC) target, maximum injection time (IT), and mass range were 70,000 (at *m*/*z* 200), 1 × 10^6^, 80 ms, and *m*/*z* 100–1500, respectively, while ddMS2 conditions were set to resolution 17,500 (at *m*/*z* 200), AGC target 1 × 10^5^, max. IT 50 ms, isolation window 2.0 *m*/*z*, and stepped normalized collision energy (NCE) of 20, 40, and 70. The quantitation of phytochemicals in the extracts was performed using a Full MS/SIM scan experiment. The method parameters were set as follows: resolution 70,000 FWHM, AGC target 3 × 10^6^, max IT 200 ms, mass range *m*/*z* 100–1500. Xcalibur ver. 4.0 and FreeStyle (Thermo Fisher Scientific, Inc., Waltham, MA, USA) ver. 1.8 SP1 was used for data acquisition and processing, respectively.

The content of each phytochemical was calculated as hyperoside equivalents using the respective quantifier ion and a 20.0 ppm isolation window. The calibration equation for hyperoside was *Y* = −130896 + 46257 × *X* (R^2^ = 0.9998), with a linear range of 15.9–509.0 ng/mL. The validation procedure for hyperoside quantification was reported previously by Marinov et al. [115]. For sample preparation, 1 mL of each extract was subjected to solid-phase extraction (Phenomenex Strata^®^ C18-E, 55 μm, 70 Å, 200 mg/3 mL) and washed three times with 1 mL of water. The analytes were eluted with methanol (4 × 1 mL), and the final volume was adjusted to 5.0 mL with methanol. An aliquot of 2 μL from each sample was injected in triplicate.

### 4.4. Chemicals

The RPMI 1640 medium used for cultivating lymphocytes was sourced from Sigma-Aldrich in Steinheim, Germany. The fetal calf serum was acquired from Sigma-Aldrich in São Paulo, Brazil. Phytohaemagglutinin (PHA) and cytochalasin B were obtained from Sigma-Aldrich in Jerusalem, Israel. Acridine orange, potassium chloride (KCl), and glacial acetic acid were purchased from Sigma-Aldrich Chemie GmbH, a subsidiary of Merck, in Steinheim, Germany. Colchicine was supplied by Merck in Darmstadt, Germany. A 0.9% sodium chloride (NaCl) solution, along with the gentamicin, was sourced from Sopharmacy, Sofia, Bulgaria. The positive control utilized in the cytogenetic experiments was the standard mutagen N-methyl-N’-nitro-N-nitrosoguanidine (MNNG) at a concentration of 50 µg/mL (CAS No.: 70-25-7). This compound was obtained from Selleck Chemicals GmbH, located in Cologne, Germany.

### 4.5. Design of Cytogenetic Experiments

Cytogenetic analyses were performed using three different test systems to assess the cytotoxic and genotoxic effects of rose extracts on barley seed meristems, ICR strain albino mice, and human lymphocyte cultures. The concentrations used in the experiments were chosen previously. Below is an outline of the experimental procedures and treatment schemes employed in the study.

#### 4.5.1. Plant Test System In Vivo

This experiment utilized root tip meristem cells derived from the reconstructed karyotype MK 14/2034 of *Hordeum vulg* are. These cells represent an exemplary model of normal cellular behavior.

The experimental procedure began with the immersion of seeds in tap water for one hour, followed by a 19-h germination period under controlled conditions in Petri dishes placed on moist filter paper at a temperature of 24 °C. Barley seeds were subjected to varying concentrations of the two rose extracts (6, 14 and 20%) for durations of either 1 h or 4 h. For comparison purposes, a positive control group received treatment with 50 µg/mL of the standard mutagen, N-methyl-N’-nitro-N-nitrosoguanidine (MNNG), for 1 h.

To assess chromosome aberrations (CA), seeds were exposed for a duration of two hours to a 0.025% colchicine solution saturated with α-bromonaphthalene, following a recovery period of 18 to 30 h, and assessed at three-hour intervals (24 °C). The root tips were subsequently fixed in a mixture of ethanol and glacial acetic acid (3:1), hydrolyzed in 1 N HCl at 60 °C for nine minutes, then Feulgen-stained, macerated in a 4% pectinase solution in distilled water for 14 min, and finally prepared for examination on slides.

The first mitoses occurring post-treatment were analyzed to determine the percentage of metaphases exhibiting CA, including chromatid and isochromatid breaks, chromatid translocations, intercalary deletions, and duplications or deletions. This analysis was conducted on a minimum of 1500 cells at various time points: 18, 21, 24, 27, and 30 h subsequent to treatment.

In the evaluation of micronuclei (MN) frequency, colchicine treatment was excluded. The incidence of MN was determined 30 h subsequent to treatment.

#### 4.5.2. Animal Test System In Vivo

Cytogenetic and cytotoxic experiments were carried out on eight-week-old male and female ICR albino mice (average body weight 20.0 ± 1.5 g), which were sourced from the Slivnitza animal breeding house, Bulgarian Academy of Sciences, Sofia. The mice were transported to the Institute of Biodiversity and Ecosystem Research’s animal facility and housed for several days under standard laboratory conditions—temperature 20–22 °C, a 12-h light/dark cycle (7 am to 7 pm), with free access to food and water.

All animal experiments were conducted in accordance with Bulgaria’s Directorate of Health Prevention and Humane Behaviour toward Animals, under Ethics Approval Protocol No. 2, 355/RD-08 (approved on 10 April 2005), and were performed only after the approval date.

The mice were randomly allocated into four groups, each comprising eight males and eight females, and housed separately to avoid cross-contamination. All tested substances were administered via a single intraperitoneal (ip) injection at a dose of 0.01 mL per gram of body weight.

The experimental groups (n = 16: 8 males and 8 females) included: Group 1—*R. damascena* SWE at an 11% solution concentration (0.01 mg/mL), Group 2—*R. damascena* SWE at a 20% solution concentration (0.01 mg/mL), Group 3—50 μg/mL MNNG (0.01 mg/mL), and Group 4—the control group, receiving only 0.9% NaCl (0.01 mg/mL) under identical conditions. The same set of experimental groups was allocated for the *R. alba* SWEs with the same concentrations. Animals were monitored twice daily for signs of toxicity after treatment.

Cytogenetic analysis for chromosome aberrations was performed at either the 24th or the 48th hour after administration of the test substances (4 males and 4 females per time point) [116]. Colchicine (0.04 mg/g body weight) was administered one hour before bone marrow collection to arrest mitosis. Blood smears were prepared prior to colchicine injection to evaluate for the presence of micronuclei. Mice were humanely euthanized using diethyl ether anesthesia. Bone marrow was flushed from the femur, then hypotonized in 0.075 M KCl at 37 °C for 15 min. Cells were fixed in cold methanol and glacial acetic acid (3:1), then dropped onto chilled slides and air-dried. Slides were stained with 5% Giemsa solution (Sigma Diagnostic).

The in vivo mammalian erythrocyte micronucleus test was performed using the micronucleus assay following OECD Test Guideline No. 474 for chemical testing [117]. The PCE micronuclei test in mouse cells has been suggested as a quick and dependable method for detecting genotoxicity caused by clastogenic and aneugenic chemical compounds [109,118]. Peripheral blood samples were collected from all dose groups 48 h after the initial rose SWEs treatment. A 5 µL sample of peripheral blood was taken from the tail vein, diluted with 45 µL of Sörensen’s phosphate buffer (pH 6.8), and a drop of the mixture was spread onto a microscope slide. The slides were air-dried, coded and fixed for 10 min using absolute methanol. The smeared preparations were stained with Acridine orange (AO) and examined under a fluorescent microscope (Axio Scope A1—Carl Zeiss) at 400× magnification, using a FITC 495 nm excitation filter (Jenoptik, Jena, Germany). The criteria for identifying micronuclei and distinguishing them from artefacts in the cytoplasm were described by Schmid [119]. Only monolayers without overlapping cells were chosen for examination on each slide.

#### 4.5.3. Human Lymphocytes In Vitro

Lymphocyte cultures were prepared from peripheral venous blood samples taken from healthy, non-smoking, non-drinking donors aged 33 to 40 years (men and woman), who were not on medication. Each culture consisted of 0.5 mL lymphocyte suspension, 3.5 mL RPMI 1640 medium, 12% heat-inactivated fetal bovine serum, 40 mg/mL gentamycin, and 0.1% mitogen-phytohemagglutinin PHA, and was incubated at 37 °C.

Chromosomal aberrations were analyzed using the standard cytogenetic method described by Evans [120]. Lymphocyte cultures were exposed to rose extract at concentrations of 6%, 11%, 14%, and 20% for 4 h, administered at the 18th hour after PHA stimulation (G1 phase). After treatment, cells were rinsed with fresh medium and cultured again at 37 °C. At 72 h, samples were prepared by adding 0.02% colchicine to arrest mitosis, then hypotonized with 0.56% KCl, fixed in methanol: acetic acid (3:1, *v*/*v*), and stained with 2% Giemsa. Untreated cells served as negative controls, while cells treated with MNNG (50 μg/mL) served as positive control.

To assess micronucleus (MN) induction, cytochalasin-B (6 μg/mL) was added 44 h after PHA stimulation, following the cytokinesis-block micronucleus (CBMN) protocol of [121]. At 72 h post-PHA stimulation, cultures were centrifuged, hypotonized with 0.56% KCl, fixed in methanol and acetic acid (3:1), dropped onto slides, and stained with 2% Giemsa solution.

All procedures conformed to the Declaration of Helsinki (2013 revision) and were approved by the Institute of Biodiversity and Ecosystem Research’s Ethics and Academic Unity Commission (No. 1, 18 February 2022). All donors gave written informed consent.

### 4.6. Cytogenetic Endpoints

#### 4.6.1. Cytotoxicity Endpoints

- Mitotic Index (MI)

The MI value indicates the number of metaphases observed per 1000 cells in each experimental variant. It serves as an indicator of cytotoxicity in both plant and lymphocyte test systems. The MI is calculated by dividing the number of metaphases counted (A) by 1000, represented by the formula: MI = A/1000. For animal cells, the MI was determined by counting the dividing cells within a sample of 1500 cells per animal [122]. In experiments involving barley and human lymphocytes, a total of 3000 cells were examined per variant to ascertain the MI. Abnormalities and MI frequencies in mice were evaluated on an individual basis for each animal, after which the mean and standard deviation were computed for each variant.

- PCE/(PCE + NCE) ratio and nuclear division index (NDI)

Additionally, the cytotoxicity of the rose extracts was assessed by measuring the PCE/(PCE + NCE) ratio in each treated ICR mouse. PCE stands for polychromatic erythrocytes, while NCE indicates normochromatic erythrocytes. For each slide, a minimum of 2000 PCEs, or a total of 4000 per animal, were analyzed to determine the frequency of micronucleated immature erythrocytes (MNPCE).

In human lymphocytes, the cytotoxicity of the tested substances was assessed by measuring the nuclear division index (NDI) through a micronuclei induction test. The calculation used the formula: (N1 + 2N2 + 3N3 + 4N4)/N, where N1–N4 are the counts of cells containing 1 to 4 nuclei, and N is the total number of cells evaluated.

#### 4.6.2. Genotoxicity Endpoints

- Assessment of Chromosomal Aberrations (CA) Induction

To evaluate the genotoxic effects of the rose extracts across all three test systems, the percentage of metaphases with chromosomal aberrations (MwA% ± SD) was calculated. In barley and lymphocyte cultures, specific aberration types—chromatid breaks (B’), isochromatid breaks (B’’), translocations (T), and intercalary deletions (D)—were identified. The main types of chromosome abnormalities, such as breaks, fragments, and centromere fusions (c/c), were scored separately for each animal across all eight experimental groups.

More than 6000 cells were analyzed in the barley and human lymphocyte tests, while 50 well-dispersed metaphases were examined from each mouse.

The plant chromosomes of the reconstructed barley karyotype MK14/2034 were analyzed to identify DNA segments more susceptible to the tested rose extract samples and/or the mutagen, using “aberration hot spots” To understand how aberrations develop in different regions, the metaphase chromosomes of H. vulgare were divided into 48 approximately equal segments. These segments were numbered according to their positions in the standard karyotype [123,124].

- Assessment of Micronuclei induction (MN)

The percentage of micronuclei (MN% ± SD) was determined for each treatment variant by counting 3000 to 6000 barley and/or human lymphocyte cells.

In the in vivo animal testing system, each peripheral blood slide was examined for the presence of micronucleated immature erythrocytes (MNPCE), with a minimum of 2000 polychromatic erythrocytes (PCE) or 4000 per animal being analyzed. To find the ratio of immature erythrocytes among all erythrocytes (both immature + mature), at least 1000 normochromatic erythrocytes (NCE) per slide or 2000 NCE per animal were counted.

### 4.7. Statistical Analysis

Experiments were conducted in triplicate. A one-way ANOVA followed by a two-tailed Fisher’s exact test, was used to analyze the treatment variants, utilizing Microsoft Excel 2010. Significance levels were defined as: *p* > 0.05 (not significant), * *p* < 0.05 (significant), ** *p* < 0.01 (more significant), and *** *p* < 0.001 (highly significant). Protocols outlined by Rieger et al. [125] and Jovtchev et al. [124] were used to identify aberration hotspots on the reconstructed barley karyotype.

## 5. Conclusions

The present investigation shows that subcritical water extracts (SWEs) of *R. damascena* and *R. alba* possess a favorable safety profile across plant, murine, and human lymphocytes test systems within a specific concentration range at working doses. Both extracts were non-cytotoxic at concentrations up to 14%. At 20%, *R. damascena* caused a mild, transient reduction in mitotic activity in mouse bone marrow at 24 h, which resolved by 48 h, whereas *R. alba* showed no such suppression. Indicators of genotoxicity (chromosomal aberrations, micronuclei) showed a slight increase with concentration in barley and human lymphocytes. *R. alba* SWE exhibited stronger genotoxic responses than *R. damascena* at equivalent doses. Nonetheless, no clastogenic or micronucleus effects were observed in vivo in mouse bone marrow. These findings align with the distinct SWE phytochemical profiles identified here for *R. damascena* and *R. alba*, indicating species-dependent phenolic distribution that underpins the modest differences in cytotoxic and genotoxic responses across assays. These species-specific profiles warrant further investigation to define the phytochemical mechanisms and their implications for human health applications.

## Figures and Tables

**Table 1:** Chromatographic profile of main compounds in the *R. damascena* and *R. alba* SWEs, performed by UHPLC-HRMS/MS analysis and calculated as hyperoside.

					SWE	*R. damascena*	SWE	*R. alba*
№	RT	Name	Quantifier Ion	Ion Type	µg/mL ± SD	Relative %	µg/mL ± SD	Relative %
1	5.98	Catechin	289.0707	[M−H]^−^	0.27 ± 0.02	0.05	5.53 ± 0.45	1.36
2	8.18	Epicatechin	289.0707	[M−H]^−^	0.11 ± 0.01	0.02	2.11 ± 0.17	0.52
3	8.89	Phenylethyl- hexpent	461.1654	[M+HCOO]^−^	12.71 ± 0.25	2.47	10.03 ± 0.56	2.47
4	9.20	2-phenylethanol O-hex	329.1231	[M−HCOO]^−^	112.94 ± 0.14	21.97	60.58 ± 1.56	14.92
5	9.32	Phenylethyl- hexpent	461.1654	[M+HCOO]^−^	2.68 ± 0.20	0.52	1.94 ± 0.10	0.48
6	9.8	Phenylethylhex- pent	461.1654	[M+HCOO]^−^	7.70 ± 0.18	1.50	5.93 ± 0.44	1.46
7	12.08	Quercetingalloyl-hexoside	615.0981	[M−H]^−^	2.55 ± 0.12	0.50	2.55 ± 0.05	0.63
8	12.32	Quercetingalloyl- hexoside	615.0981	[M−H]^−^	1.75 ± 0.04	0.34	1.03 ± 0.06	0.25
9	12.60	Ellagicacid	300.9979	[M−H]^−^	60.86 ± 1.01	11.84	21.88 ± 0.60	5.39
10	12.73	Rutin	609.1450	[M−H]^−^	4.80 ± 0.28	0.93	4.17 ± 0.21	1.03
11	12.85	Hyperoside	463.0871	[M−H]^−^	29.05 ± 0.35	5.65	30.63 ± 0.6	7.55
12	13.15	Isoquercitrin	463.0871	[M−H]^−^	27.51 ± 0.48	5.35	27.20 ± 0.52	6.70
13	13.88	Quercetingalloyl-hexoside	615.0981	[M−H]^−^	1.27 ± 0.10	0.25	2.10 ± 0.18	0.52
14	14.17	Quercetin HMG-O-hexoside	607.1294	[M−H]^−^	2.04 ± 0.14	0.40	2.31 ± 0.21	0.57
15	14.25	Kaempferol-3-O-galactoside	447.0922	[M−H]^−^	16.86 ± 0.10	3.28	17.87 ± 0.36	4.40
16	14.44	Kaempferol-hexoside- methylpentoside	593.1528	[M−H]^−^	12.33 ± 0.07	2.40	11.43 ± 0.16	2.82
17	14.52	Quercetinpentoside	433.0765	[M−H]^−^	8.07 ± 0.43	1.57	12.16 ± 0.36	3.00
18	14.61	Quercetinhex-deoxyhexoside	609.1450	[M−H]^−^	7.64 ± 0.55	1.49	7.66 ± 0.58	1.89
19	14.74	2-phenylethanol ester of galoylhexoside	435.1303	[M−H]^−^	23.56 ± 0.20	4.58	17.13 ± 0.19	4.22
20	14.90	Kaempferol-3-O-glucoside	447.0922	[M−H]^−^	83.57 ± 0.26	16.26	82.12 ± 1.28	20.23
21	15.94	Kampferol-3-O-xyloside	417.0836	[M−H]^−^	6.74 ± 0.03	1.31	8.10 ± 0.05	2.00
22	16.48	Kampferol-3-O-arabinoside	417.0836	[M−H]^−^	13.39 ± 0.13	2.60	18.87 ± 0.25	4.65
23	16.72	Kaempferol-hexoside- methylpentoside	593.1528	[M−H]^−^	13.50 ± 0.17	2.63	13.02 ± 0.28	3.21
24	17.17	Kaempferol-3-O-rhamnoside	431.0991	[M−H]^−^	14.59 ± 0.04	2.84	15.70 ± 0.20	3.87
25	17.7	Quercetinacetyl-hex- deoxyhexoside	651.1556	[M−H]^−^	2.23 ± 0.10	0.43	2.82 ± 0.12	0.69
26	19.49	Quercetin	301.0343	[M−H]^−^	11.58 ± 0.63	2.25	0.70 ± 0.02	0.17
27	19.68	Kaempferolacetyl-hex- deoxyhexoside	635.1607	[M−H]^−^	8.12 ± 0.58	1.58	11.53 ± 0.38	2.84
28	21.54	Citronellolhex-pent	495.2436	[M+HCOO]^−^	9.22 ± 0.21	1.79	5.96 ± 0.23	1.47
29	22.1	Citronellolhex-pent	495.2436	[M+HCOO]^−^	3.52 ± 0.25	0.68	2.31 ± 0.12	0.57
30	23.24	Kaempferol	285.0399	[M−H]^−^	12.88 ± 0.48	2.51	0.57 ± 0.02	0.14

Rt—retention time.

## Data Availability

The manuscript presents all the data collected from this research.

## Abbreviations

The following abbreviations are used in this manuscript:

SWE	Subcritical water extraction
UHPLC-HRMS/MS	Ultra-High-Performance Liquid Chromatography coupled to High-Resolution Tandem Mass Spectrometry
MI	Mitotic index
SWEs	Subcritical water extracts
ROS	Reactive oxygen species
PCE	Polychromatic erythrocytes
NCE	Normochromatic erythrocytes
MNPCE	Micronucleated immature erythrocytes
NDI	Nuclear Division Index
MN	Micronuclei
CA	Chromosomal aberration
B”	Isochromatid break
B’	Chromatid break
T	Translocation
MNNG	N-nitro-N’-nitro-N-nitrosoguanidine
OECD	Organisation for Economic Co-operation and Development
